# ADAD1 and ADAD2, testis-specific adenosine deaminase domain-containing proteins, are required for male fertility

**DOI:** 10.1038/s41598-020-67834-5

**Published:** 2020-07-14

**Authors:** Elizabeth Snyder, Lauren Chukrallah, Kelly Seltzer, Leslie Goodwin, Robert E. Braun

**Affiliations:** 1 Rutgers University, The State University of New Jersey, New Brunswick, NJ USA; 2 The Jackson Laboratory, Bar Harbor, Maine, USA

**Keywords:** Spermatogenesis, RNA editing, Reproductive biology

## Abstract

Adenosine-to-inosine RNA editing, a fundamental RNA modification, is regulated by adenosine deaminase (AD) domain containing proteins. Within the testis, RNA editing is catalyzed by ADARB1 and is regulated in a cell-type dependent manner. This study examined the role of two testis-specific AD domain proteins, ADAD1 and ADAD2, on testis RNA editing and male germ cell differentiation. ADAD1, previously shown to localize to round spermatids, and ADAD2 had distinct localization patterns with ADAD2 expressed predominantly in mid- to late-pachytene spermatocytes suggesting a role for both in meiotic and post-meiotic germ cell RNA editing. AD domain analysis showed the AD domain of both ADADs was likely catalytically inactive, similar to known negative regulators of RNA editing. To assess the impact of *Adad* mutation on male germ cell RNA editing, CRISPR-induced alleles of each were generated in mouse. Mutation of either *Adad* resulted in complete male sterility with *Adad1* mutants displaying severe teratospermia and *Adad2* mutant germ cells unable to progress beyond round spermatid. However, mutation of neither *Adad1* nor *Adad2* impacted RNA editing efficiency or site selection. Taken together, these results demonstrate ADAD1 and ADAD2 are essential regulators of male germ cell differentiation with molecular functions unrelated to A-to-I RNA editing.

## Introduction

RNA editing is a class of post-transcriptional modification that enhances the complexity of the transcriptome^1^. On a molecular level, RNA editing is the irreversible chemical modification of a nucleotide within an intact RNA. Two basic types of RNA editing are observed in mammals, adenosine to inosine and cytosine to uridine, of which adenosine to inosine (A-to-I) occurs much more frequently^2^. A-to-I RNA editing may occur at one or more sites in a given target RNA and across the entire population of a target RNA or a fraction thereof. To date, A-to-I RNA editing has been observed in a diverse range of RNAs including mRNAs, small RNAs, and long non-coding RNAs^3,4^. Functionally, inosine mimics the behavior of guanine and is read as such by the translational machinery^5^, thus A-to-I RNA editing events behave as A-to-G mutations on the RNA level. As a consequence, the outcome of A-to-I RNA editing varies widely based on the RNA target and the edited site or sites within the target. Reported impacts of RNA editing include altered protein coding potential^6^, splicing patterns^7^, and microRNA recognition (either from edits within miRNAs^8^ themselves or their targets^2^). The physiological relevance of RNA editing is clear as animals deficient for A-to-I RNA editing enzymes often show severe physiological defects^9–11^.

In mammals, RNA editing is catalyzed by two adenosine deaminase (AD) domain-containing proteins: Adenosine Deaminase, RNA-specific 1 and 2 (ADAR1 and ADAR2 in the human, and ADAR and ADARB1 in the mouse, respectively). Both enzymes contain at least one double-stranded RNA binding motif and an AD domain, which directly catalyzes the conversion of adenosine to inosine^5^. Within the AD domain, four amino acids coordinate a zinc in the active site of the domain and are presumably required for catalytic activity^12^. This presumption is further supported by the observation that all four amino acids are conserved in the single catalytically active RNA editing enzyme of *C. elegans*, cADR-2^12^. AD domain activity also relies on binding of inositol hexakisphosphate which is coordinated by a large number of amino acids likewise largely conserved in cADR-2.

In mammals and worms, RNA editing is further regulated by catalytically inactive AD domain proteins: ADAR3 in humans (ADARB2 in mice) and cADR-1 in *C. elegans*. In humans ADAR3 negatively regulates ADAR1 and ADAR2-mediated RNA editing^13^ via competitive binding of target RNAs^14^ while in *C. elegans*, cADR-1 regulates the activity of cADR-2 by facilitating cADR-2′s interaction with target RNAs via a protein–protein interaction^15^. Although it is unclear why ADAR3 is catalytically inactive given its AD domain contains all of the identified amino acids necessary for editing activity, mutations at each of the zinc coordinating amino acids in cADR-1 suggests mutations at known cofactor binding sites may indicate AD domains that regulate, as oppose to catalyze, RNA editing.

A-to-I RNA editing efficiency (the fraction of a given target RNA edited) and frequency (the number of editing sites within the transcriptome) is highly tissue and cell-type dependent. However, this variability cannot be explained by catalytically active RNA editing enzyme expression^16^ suggesting tissue or cell-specific factors may play extensive roles in regulating tissue-specific levels of RNA editing. A large-scale analysis in humans identified two such tissue-specific regulatory systems^13^. In the brain, which displays unusually high levels of RNA editing, the expression of catalytically active ADARs (ADAR1 and ADAR2) was able to explain the majority of region-specific editing levels only when combined with expression of the negative RNA editing regulator ADAR3. Similarly, in skeletal muscle the expression of AIMP2, an aminoacyl-tRNA synthetase, was directly correlated with the unusually low levels of RNA editing detected therein. Other tissue-specific mechanisms of RNA editing regulation specifically by RNA binding remain to be identified.

One potentially fruitful approach to the identification of other RNA editing regulatory mechanisms is the identification of tissue-specific AD domain proteins. In the current mouse genome, a total of ten AD domain proteins that can act on RNA are annotated (USCS Genome Browser). Of these, one (Adenosine deaminase domain containing protein 1, testis specific—*Adad1*) has previously been identified as tissue-specific, being expressed exclusively in the testis^17^ and required for normal male fertility^18^. As such, ADAD1 is a good candidate for tissue-specific regulation of RNA editing. Further, RNA editing in the testis is somewhat unusual in that, unlike many tissues where ADAR and ADARB1 act in concert to regulate RNA editing, testis RNA editing it is driven exclusively by ADARB1 ^19^. Moreover, *Adarb1* expression in specific cell populations of the testis does not correlate directly to the level of cell-specific RNA editing observed. The adult testis is composed of two cell populations: the developing germ cells and the somatic cells that support their differentiation. As germ cells mature (undergo spermatogenesis), they transition through three basic phases: mitosis, meiosis, and post-meiotic differentiation (referred to as spermiogenesis). These developmental stages are associated with dramatic changes in the transcriptome^20^ and RNA regulation^21^. In the case of RNA editing, *Adarb1* has relatively high expression in the mitotic spermatogonia and post-meiotic spermatids and much lower expression in the meiotic spermatocytes and Sertoli cells, the dominant somatic cell population^19^. This is in contrast to the number of RNA editing sites detected in these populations where very limited sites are observed in spermatogonia and spermatids, a moderate number of sites detected in spermatocytes, and many sites detected in Sertoli cells. In sum, these observations suggest additional layers of RNA editing regulation within the testis^19^.

Herein, we describe the identification of a novel testis-specific ADAD1-related AD domain protein, ADAD2, and test the impact of *Adad1* and *Adad2* mutation on testicular RNA editing via CRISPR-induced mutation in mice. Further, we quantify the effect of *Adad1* and *Adad2* mutation on germ cell differentiation and male fertility. These findings provide insight into the potential functions of AD domain containing proteins in the male germ cell.

## Results

### ADAD1 and ADAD2 are testis-specific adenosine deaminase domain containing proteins

ADAD1 (also known as TENR) is a previously described RNA binding protein^17^ that contains a double-stranded RNA binding motif (dsRBM) and AD domain very similar to the ADARs (Supp. Figure 1A and B), suggesting it may regulate RNA editing. To determine if additional AD domains were encoded in the mouse genome, the current mouse gene annotation was queried for AD domain containing proteins, which revealed a second *Adad* gene, *Adad2*, that appeared to encode a protein with similar domains to ADAD1. Careful examination of both AD domains showed ADAD1 and ADAD2 have mutations in three of the four zinc coordinating residues required for AD catalytic activity. These mutations are similar to those observed in the catalytically inactive, RNA editing regulatory protein, cADR-1, suggesting the hypothesis that ADAD1 and ADAD2 may regulate RNA editing catalyzed by ADARs. This hypothesis is further supported by a recent report demonstrating the dsRBM of ADAD2 has a preference for structured dsRNAs similar to known ADAR substrates^22^.

**Figure 1:**
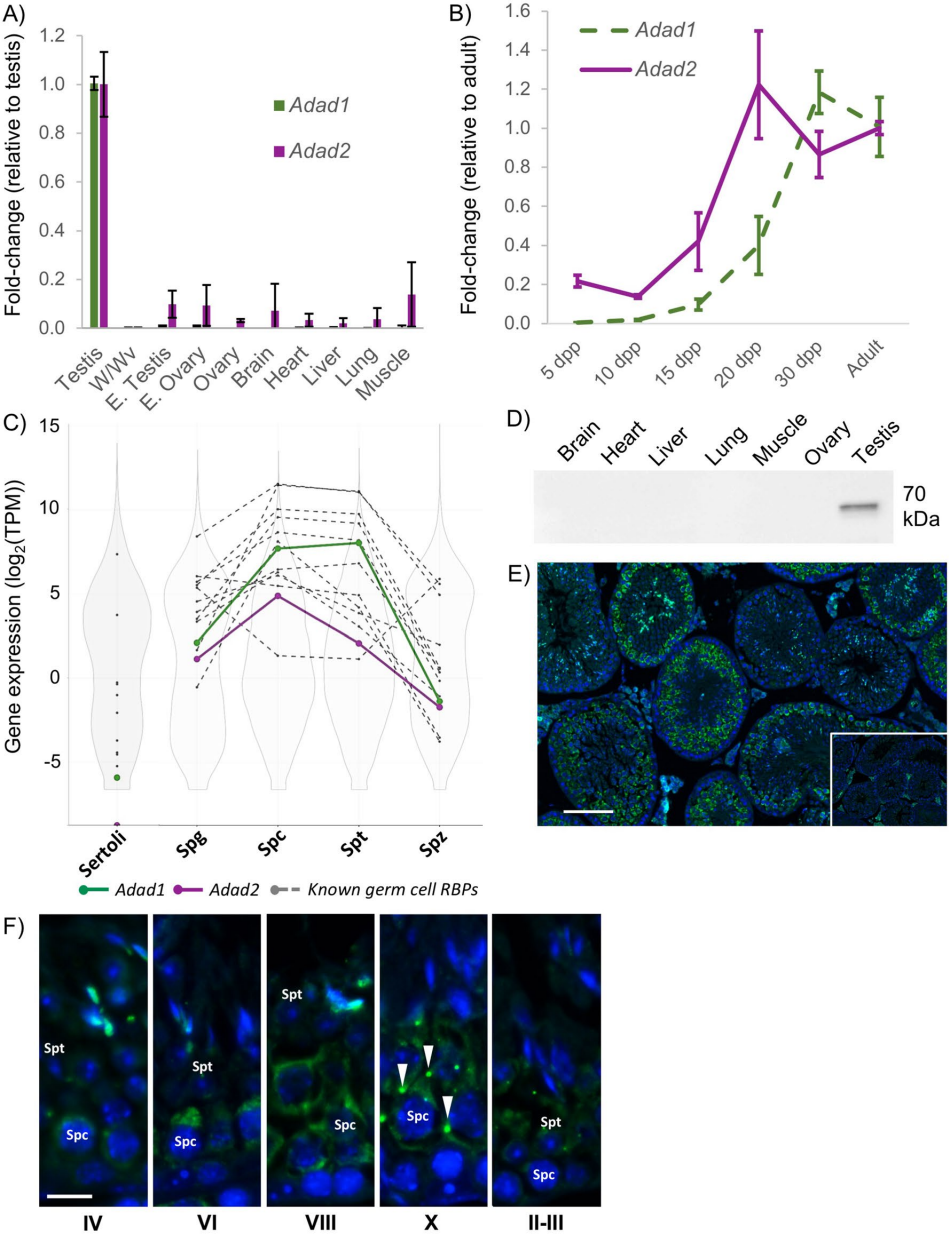
ADAD1 and ADAD2 are testis-specific adenosine deaminase domain containing proteins. (**A**) *Adad1* and *Adad2* expression as assessed by qRT-PCR across a panel of adult and embryonic wildtype and selected mutant tissues. W/Wv—adult testis from W/Wv males. E—embryonic, 15.5 days post-coitum. (**B**) qRT-PCR of *Adad1* and *Adad2* expression throughout testis development. dpp—days post-partum. N ≥ 3 per sample, error bars—standard deviation. (**C**) Expression of *Adads* derived from RNA-sequencing of isolated testicular somatic (Sertoli) and germ cells (23) relative to all expressed genes (violin plots—grey and white) and select genes encoding known male germ cell RNA binding proteins (line and dot plots—grey). Spg—spermatogonia, Spc—spermatocytes, Spt—round spermatids, Spz—spermatozoa. (**D**) Western blotting of ADAD2 across a panel of adult wildtype tissue. Similar results obtained for both ADAD2 antibodies, representative blot shown, confirmation of equal protein loading reported in Supp. Figure 3. (**E**) Localization of ADAD2 by immunofluorescence in adult mouse testis. Similar results obtained for both ADAD2 antibodies, representative images shown. Inset: no-primary antibody control. Bar—100 um. (**F**) ADAD2 spermatocyte localization by seminiferous tubule cross section stage. Arrow heads—perinuclear granules, bar—10 um. Spc—spermatocytes, Spt—round spermatids.

Previous work^17^ demonstrated testis-specific expression of *Adad1*, however no detailed in vivo analysis of *Adad2* has been reported. To determine whether *Adad2* was likewise expressed in a tissue-dependent manner, quantitative RT-PCR was performed across a panel of wildtype and mutant mouse tissues. This analysis demonstrated both *Adad1* and *Adad2* are expressed predominantly in the testis (Fig. 1A). Further, lack of expression in embryonic testes, which do not contain mature germ cells, and adult testes from a mutant model in which germ cells fail to develop (W/Wv), suggested both *Adad1* and *Adad2* expression is derived primary from differentiating germ cells. As the testis develops, successively more mature germ cells appear, thus analysis of gene expression across testis development serves as a proxy for analysis of expression throughout germ cell development. *Adad2* expression dramatically increases around 15 dpp (Fig. 1B) which coincides with the appearance of the mid-meiotic pachytene spermatocytes. *Adad1*, on the other hand, has a somewhat delayed expression more indicative of early post-meiotic germ cells that correlates well with previous reports demonstrating ADAD1 to be detectible primarily in round spermatids^17^. To more closely define the expression profile of the *Adad* genes, their expression was examined by RNA-sequencing of isolated testicular cell types^20^. Comparison to total expressed genes as well as genes with known roles in RNA biology (Fig. 1C, Supp. Table 1) demonstrated both *Adads* to be moderately to highly expressed in spermatocytes but only *Adad1* to have significant expression in spermatids. This expression profile suggests ADAD2 is less abundant than ADAD1 and exclusive to meiotic spermatocytes.

To determine if ADAD2 protein is enriched in or specific to meiotic cell types in the testis, two rabbit polyclonal antibodies against the C-terminal 19 amino acids of ADAD2 were generated. In both cases, Western blotting of an adult tissue panel detected a single band of approximately 70 kDa exclusive to the testis, confirming ADAD2 to be testis specific (Fig. 1D). Using these antibodies, the cellular localization of ADAD2 within the testis was determined (Fig. 1E). Immunofluorescence in adult testis showed ADAD2 to localize primarily to pachytene spermatocytes in agreement with the developmental and cellular expression profiles. Further, ADAD2 showed a developmentally-regulated sub-cellular localization pattern wherein it transitioned from diffusely cytoplasmic early in pachytene spermatocytes and coalesced into several perinuclear granules by late pachynema (Fig. 1F). These granules were dispersed early in round spermatids and ADAD2 was undetectable in round spermatids by step 4. An additional signal was detected in late elongated spermatid nuclei and cytoplasm. Given the expression profile of *Adad2*, this signal likely represents non-specific background resulting from non-affinity purified antibody. The localization of ADAD2 is different from that observed for ADAD1, which is primarily detected in round spermatids^17^, suggesting that ADAD1 and ADAD2 function at different stages of germ cell differentiation.

### CRISPR-induced mutation of *Adad1* and *Adad2* results in null alleles

**Figure 2:**
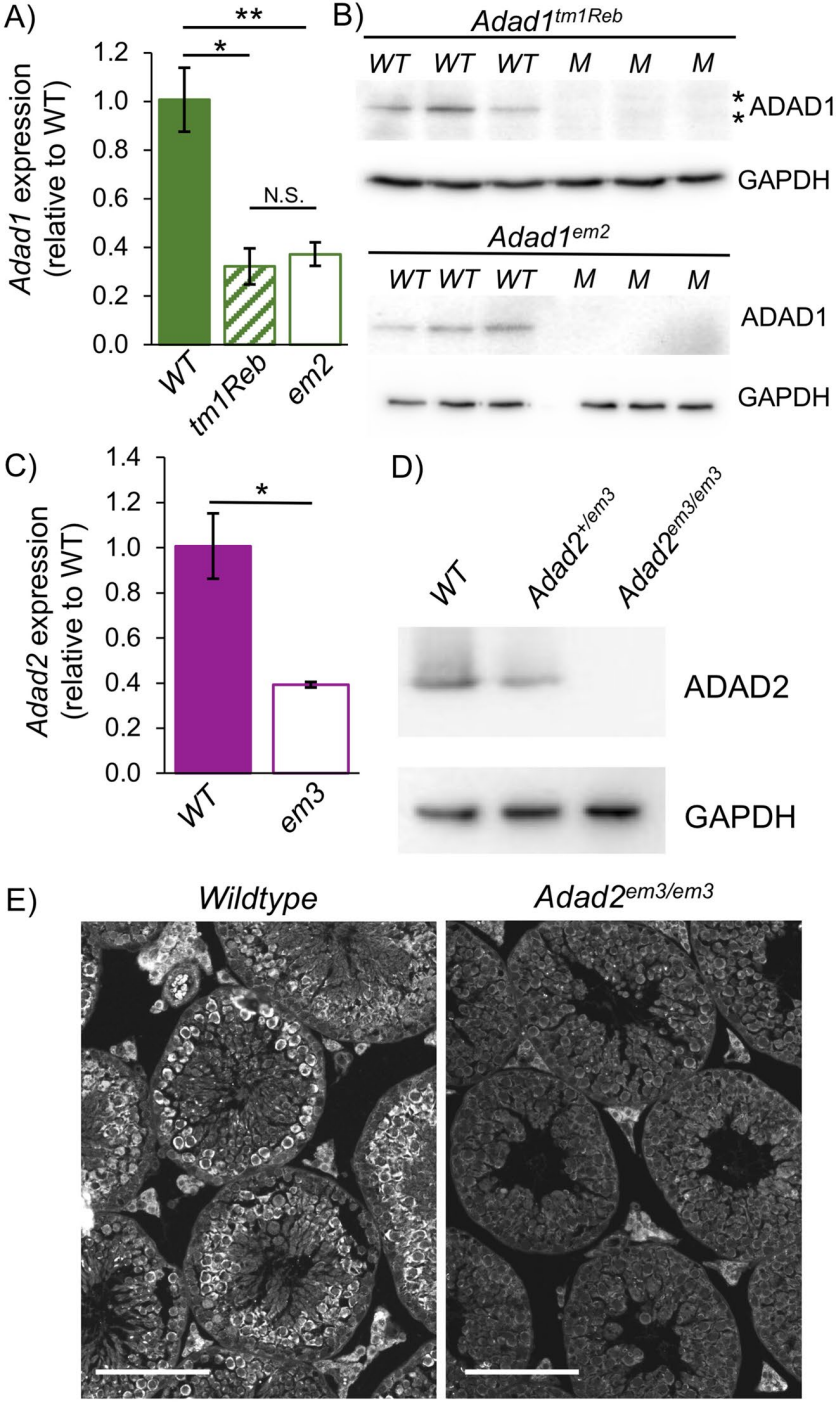
CRISPR-induced mutation results in molecular ablation of *Adad1* and *Adad2.* (**A**) *Adad1* expression in adult testes of wildtype (WT) and homozygous *Adad1* mutants assessed by qRT-PCR. N ≥ 3, error bars—standard deviation. (**B**) Western blot detection of ADAD1 (~ 70 kDa) in adult wildtype (WT) and *Adad1* mutant (M) testes. GAPDH (34 kDa) shown as a loading control. Allele of interest indicated above blot. Each lane represents an independent biological replicate. Asterisks indicate additional bands specific to the *tm1Reb* allele recognized by the ADAD1 antibody. (**C**) *Adad2* expression in wildtype (WT) and *Adad2*^*em3/em3*^ adult testes assessed by qRT-PCR. N ≥ 3, error bars—standard deviation. (**D**) Western blot detection of ADAD2 (~ 70 kDa) in adult *Adad2* wildtype (WT), heterozygous mutant, and homozygous mutant testes. GAPDH (34 kDa) shown as a loading control. **E.** ADAD2 immunofluorescence in wildtype and *Adad2* mutant adult testis. Bar—100 um. * p-value < 0.01, ** p-value < 0.001, N.S.—not significant.

To test the requirement for ADAD1 and ADAD2 during spermatogenesis, we generated CRISPR-induced global mutations in *Adad1* and *Adad2* (Supp. Figure 1C through H). In the case of *Adad1*, a previously reported allele (*Adad1*^*tm1Reb*^), generated via insertional mutagenesis that ablated the 5′ most translation start site, had been shown to be required for normal male fertility^18^. However since that time additional annotation evidence suggested an ADAD1 protein may also be generated from a more 3′ translation start site still intact in the *Adad1*^*tm1Reb*^ allele. In order to ensure total protein ablation, targeting of *Adad1* focused on the region around the more 3′ translation start site. No such constraints were required for *Adad2* as it contains only a single translation start site. CRISPR-induced mutagenesis resulted in multiple alleles for both genes, and for each gene all three alleles displayed similar molecular and reproductive phenotypes. A single allele for each was selected for further study (Fig. 2). The selected *Adad1* CRISPR allele *Adad1*^*em2(IMPC)J*^ (herein referred to as *Adad1*^*em2*^) was compared against wildtype and *Adad1*^*tm1Reb*^ whole testes on the message level by quantitative RT-PCR and both alleles showed a similar level of message reduction relative to wildtype (Fig. 2A). Given the molecular nature of the mutations (insertional mutagenesis in the case of *Adad1*^*tm1Reb*^ and an early frameshift mutation in the case of *Adad1*^*em2*^), this reduction is likely due to non-sense mediated decay of RNA generated from the mutant loci. As message was reduced but not lost entirely in both mutant models, further analysis on the protein level was conducted. Using a custom antibody derived from the C-terminal end, protein levels of ADAD1 were examined in *Adad1*^*tm1Reb*^ and *Adad1*^*em2*^ homozygous mutants (Fig. 2B). This analysis showed that there was no detectible ADAD1 protein in *Adad1*^*em2*^ testis, demonstrating it to be a true molecular null, while the antibody recognized several proteins of somewhat altered size and greatly reduced total abundance in the *Adad1*^*tm1Reb*^ model suggesting *Adad1*^*tm1Reb*^ may not entirely ablate ADAD1. Analysis of *Adad2* message (Fig. 2C) and protein in the selected *Adad2* CRISPR allele *Adad2*^*em3(IMPC)J*^(herein referred to as *Adad2*^*em3*^) demonstrated a significant reduction in message, likely driven by non-sense mediated decay of the premature stop containing mutant transcript, and a complete loss of ADAD2 in the CRISPR model, as determined by both Western blotting and immunofluorescence (Fig. 2D, E), demonstrating the specificity of the newly generated anti-ADAD2 antibody and confirming *Adad2*^*em3*^ as a genuine ADAD2 null allele.

### Two *Adad1* alleles have moderately different impacts on male germ cell differentiation

A previous mutant allele of *Adad1* had been shown to be male sterile with defects late in spermiogenesis^18^. To determine if the newly-generated *Adad1* CRISPR allele phenocopied the previous allele, detailed phenotyping was performed on both alleles in parallel. Adult testicular histology showed a complete complement of differentiated germ cells in mutant adult testis and no gross morphological defects in the testicular germ cells (Supp. Figure 2A). Careful seminiferous tubule staging identified a minor defect in spermiation (the release of mature spermatozoa from the seminiferous epithelium) in both models as indicated by retained spermatids in stages IX and X, similar to a previously reported phenotype for the *Adad1*^*tm1Reb*^ allele (Supp. Figure 2B). In the *Adad1* CRISPR mutant this defect in spermiation appears to coincide with the presence of elongated spermatids near the basal membrane of stage IX and X tubules (Supp. Figure 2C). The incidence of these aberrantly localized spermatids was highest in stage IX tubules but was also observed as late as stage XI. Further, while some spermatids were observed as single cells, the majority appeared as large clumps. These findings suggest spermatids that fail to undergo spermiation in the *Adad1* CRISPR mutant are resorbed during the late stages of spermatogenesis.

**Figure 3:**
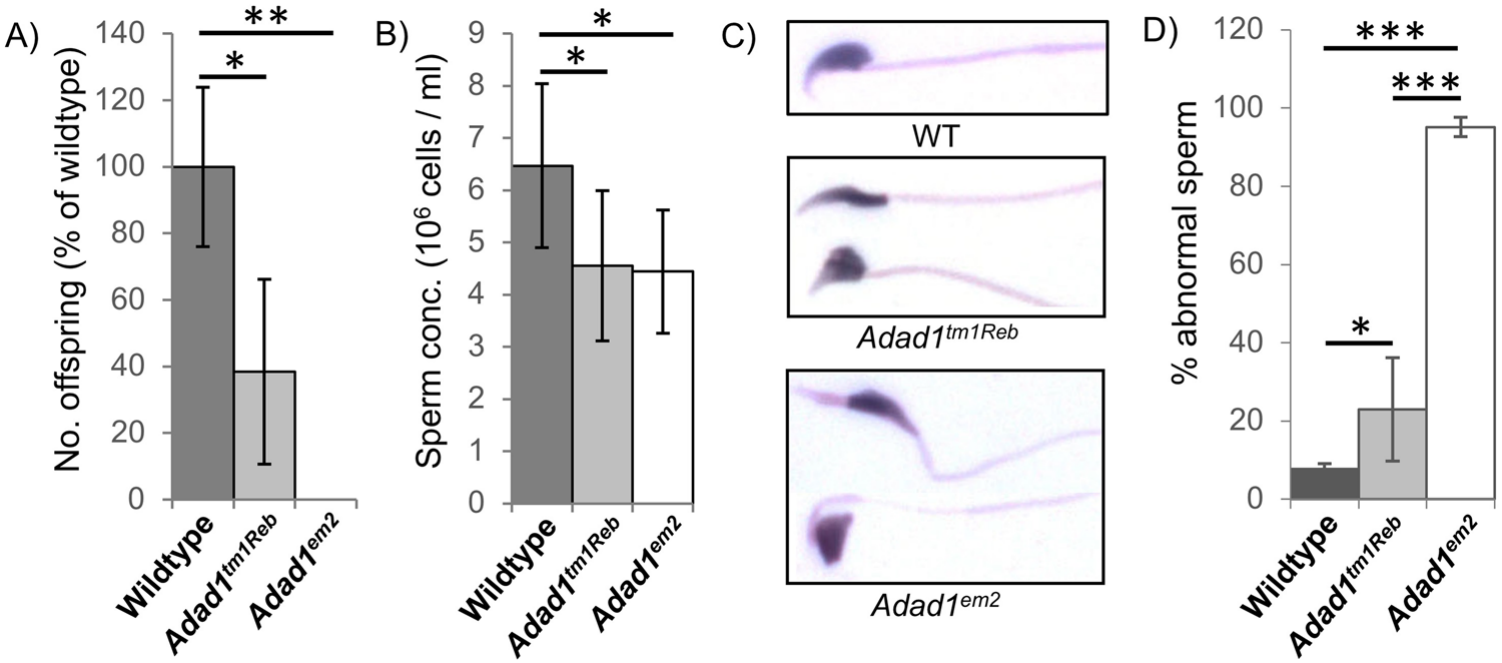
Comparative phenotypes in *Adad1*^*tm1Reb*^ and *Adad1*^*em2*^ males. Quantification of wildtype and homozygous *Adad1* mutant (**A**) offspring sired (N = 3) and (**B**) epididymal sperm concentration (N ≥ 4). (**C**) Representative comparison of mature spermatozoa morphology between wildtype, *Adad1*^*tm1Reb*^, and *Adad1*^*em2*^males. (**D**) Quantification of morphologically abnormal epididymal sperm in homozygous mutant *Adad1*^*tm1Reb*^ and *Adad1*^*em2*^ adult males. Error bars—standard deviation, * p-value < 0.05, ** p-value < 0.005, *** p-value < 0.0001.

In order to more closely examine the impact of the two *Adad1* mutant alleles on released spermatozoa, male fertility and sperm morphology were examined. Mutant males showed normal mating behavior and generated a similar number of plugged females as wildtype males. However, both *Adad1* mutant models generated fewer offspring (Fig. 3), with *Adad1*^*tm1Reb*^ males generating approximately a third of the offspring as wildtype, and *Adad1*^*em2*^ generating no offspring, again suggesting the *Adad1*^*em2*^ allele has a greater impact on germ cell differentiation than *Adad1*^*tm1Reb*^. The relative difference in fertility could not be explained as a function of sperm concentration as males homozygous for either allele had similarly reduced epididymal sperm concentration. Sperm from both mutant models displayed a range of morphology defects with the most common defect in both models being abnormally elongated or shortened heads. Overall, morphological defects of individual sperm in *Adad1*^*em2*^ appeared more severe than those in *Adad1*^*tm1Reb*^. This severity was matched in the population as a whole as the incidence of morphological defects was significantly higher in *Adad1*^*em2*^ sperm than in *Adad1*^*tm1Reb*^ sperm. Taken together, analyses of both *Adad1* alleles demonstrates ADAD1 to be important for the late stages of male germ cell differentiation.

### ADAD2 is required for normal male germ cell differentiation

**Figure 4:**
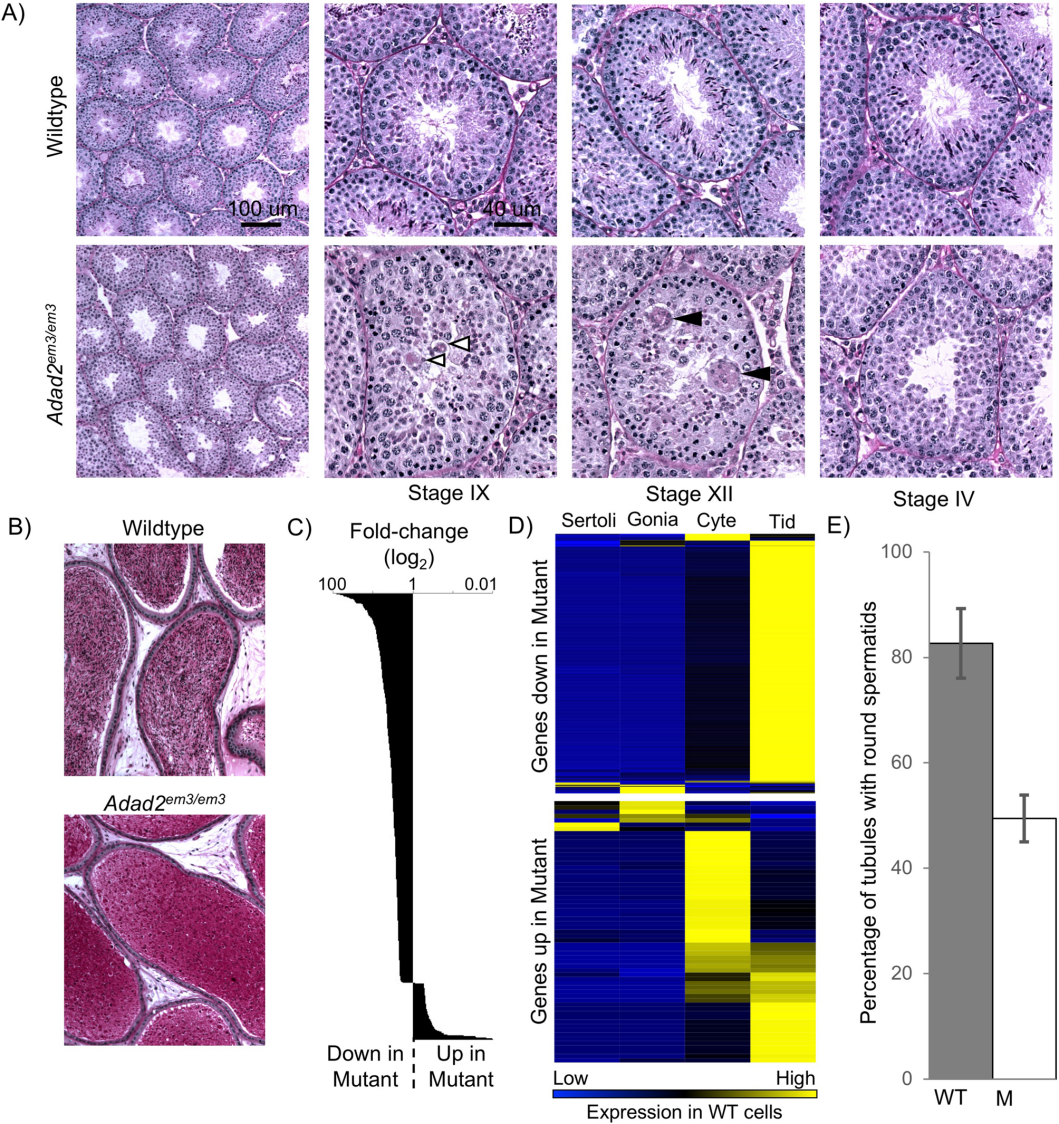
Knockout of *Adad2* results in germ cell development defects distinct from *Adad1*. (**A**) PAS-stained adult testis from wildtype and *Adad2*^*em3/em3*^ animals showing abnormal post-meiotic germ cell differentiation associated with a gradual loss of post-meiotic germ cells. Stage of the seminiferous epithelium indicated by Roman numerals. White arrowheads—abnormal elongating spermatids. Black arrows—multinucleated cells. (**B**) PAS-stained adult epididymis from wildtype and *Adad2*^*em3/em3*^ animals showing lack of spermatozoa in mutant epididymis. (**C**) Gene expression assessed in late juvenile *Adad2*^*em3/em3*^ whole testis as compared to wildtype by RNA-sequencing. (**D**) Cell-type expression profiles (derived from wildtype cells) of genes down or up-regulated in mutant testis. Data derived from^23^. (**E**) Quantification of seminiferous tubule cross-sections with round spermatids in late juvenile testes of wildtype (WT) and *Adad2*^*em3/em3*^ (M).

Prior to analysis of RNA editing, the physiological role of ADAD2 in male germ cell differentiation was assessed. Histological analysis of adult testes from *Adad2*^*em3*^ homozygous males showed morphologically abnormal post-meiotic germ cells transitioning from round to elongating spermatids (Fig. 4A) (stage IX—step 9 spermatids). Later, elongating spermatids showed clear defects with dramatically reduced numbers and what appeared to be large multinucleated cells (stage XII—step 12 spermatids), indicative of cell death^23^. This resulted in an almost total absence of elongated spermatids by stage IV and no observable spermatozoa in either *Adad2*^*em3/em3*^ testis or epididymis (Fig. 4B). Given the severity of the testicular germ cell defects and lack of mature spermatozoa in the *Adad2*^*em3/em3*^ epididymis, *Adad2*^*em3/em3*^ males are assumed to be sterile.

RNA-sequencing of late juvenile *Adad2* wildtype and mutant testes (25 dpp) was performed (Fig. 4C) to identify potential causes for germ cell loss in the mutant. This time point was selected as it is prior to the onset of distinguishable morphological defects in the mutant elongating spermatids which are normally detected approximately 30 days post-partum. Initial analysis showed the majority of significantly altered genes were down-regulated in the mutant testis relative to wildtype. To characterize the normal expression profile of genes differentially expressed in the mutant testis, their expression in wildtype isolated testicular cells was assessed (Fig. 4D) using publicly available RNA-sequencing data^24^. Of these genes, the vast majority were expressed primarily or exclusively in round spermatids within the wildtype testis. To determine if this pattern of gene expression was due to ADAD2′s molecular function or a result of cellular composition changes in the mutant testis, morphological quantification of round spermatids at 25 dpp was undertaken. This analysis showed a previously unappreciated reduction in the number of tubule cross sections containing round spermatids in the mutant relative to the wildtype (Fig. 4E) which leads to a reduction of total round spermatids in the mutant testis. From the perspective of gene expression changes, this change in cellular composition would result in reduced expression of round spermatid genes concurrent with increased expression of spermatocyte genes, a pattern clearly observed in the RNA-sequencing data. As such, any round spermatid-enriched genes up-regulated in the mutant or spermatcyte-enriched genes down regulated in the mutant would represent potential transcriptional level changes driven by ADAD2 loss. Detailed analysis identified only two genes with these particular gene expression profiles: *Wdr34*, a spermatocyte-enriched gene down-regulated in the mutant, and *Gm9*, a round spermatid gene up-regulated in the mutant. The relatively small number of genes with transcriptional changes in the mutant further suggests ADAD2 acts primarily post-transcriptionally. Taken together, these results demonstrate ADAD2 to be required for post-meiotic germ cell differentiation, likely as a function of molecular events occurring during mid- to late-meiosis.

**Figure 5:**
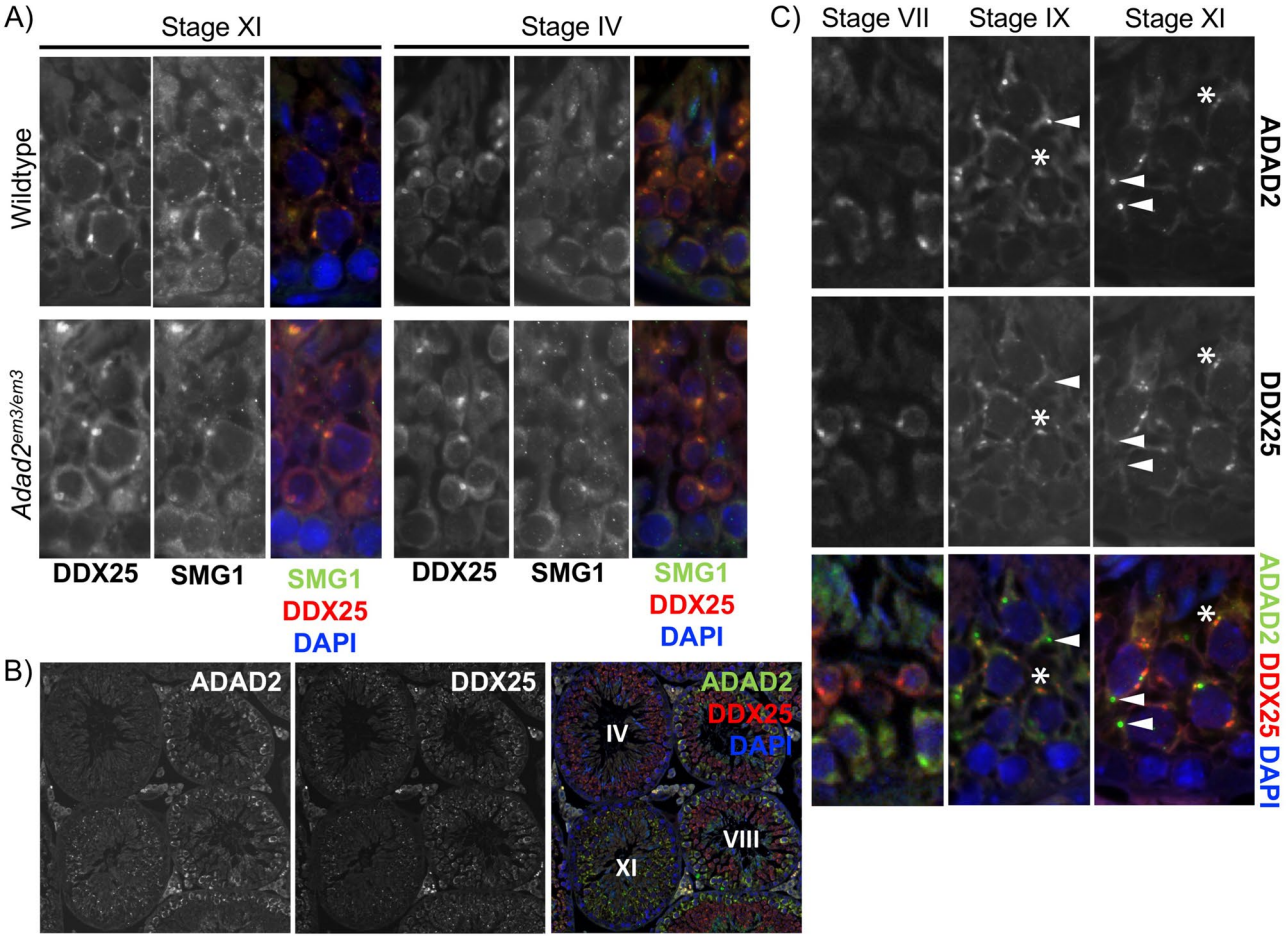
ADAD2 is not required for formation of the chromatoid body but does constitute its own, unique RNA granule. (**A**) Co-localization of the chromatoid body markers DDX25 and SMG1 by immunofluorescence in wildtype and *Adad2*^*em3/em3*^ adult testes showing similar patterns of co-localization in late pachytene spermatocytes (Stage XI) and early round spermatids (Stage IV) across both phenotypes. (**B**) Co-localization of aDAD2 and DDX25 by immunofluorescence in adult wildtype testis showing tubule to tubule variation and distinct localization patterns. Tubule stage indicated by Roman numeral. (**C**) Co-localization of ADAD2 and DDX25 in wildtype adult testis showing ADAD2 granule formation in stage VII pachytene spermatocytes, followed by moderate co-localization with DDX25 granules observed in stage IX pachytene spermatocytes. Stage XI pachytene spermatocytes showing demarcation of ADAD2 and DDX25 granules in pachytene spermatocytes. Arrowheads indicate ADAD2-only granules. Asterisks indicate ADAD2/DDX25 granules.

ADAD2′s granular localization late in meiosis and its potential impact on post-transcriptional regulation in meiotic or post-meiotic germ cells suggests a potential role in formation of the chromatoid body (CB), a post-meiotic germ cell RNA-rich granule that serves as the site for a diverse range of RNA processes^25^. To determine if ADAD2 loss impacted CB formation, two known CB proteins (DDX25^26^ and SMG1^25^) were examined by immunofluorescence in wildtype and *Adad2*^*em3/em3*^ adult testis (Fig. 5A). This analyses showed colocalization of both markers in cytoplasmic granules from late pachytene spermatocytes to round spermatids. This pattern was retained in *Adad2*^*em3/em3*^ germ cells suggesting CB formation is normal with ADAD2 loss and further suggesting ADAD2 granules may be distinct from the DDX25/SMG1-positive CB precursor granules observed in late pachytene spermatocytes. To determine if this was the case, ADAD2 was immunolocazed relative to DDX25 (Fig. 5B, C). ADAD2 granules were first observed in mid-pachytene spermatocytes and persisted until the end of meiosis. In contrast, DDX25 granules were first observed in late pachytene spermatocytes and showed limited and developmentally-dependent overlap with ADAD2 granules. Taken together, these findings demonstrate ADAD2 to be a potential post-transcriptional regulator with functions independent of chromatoid body formation.

### Neither *Adad1* nor *Adad2* mutation impacts mRNA editing

**Figure 6:**
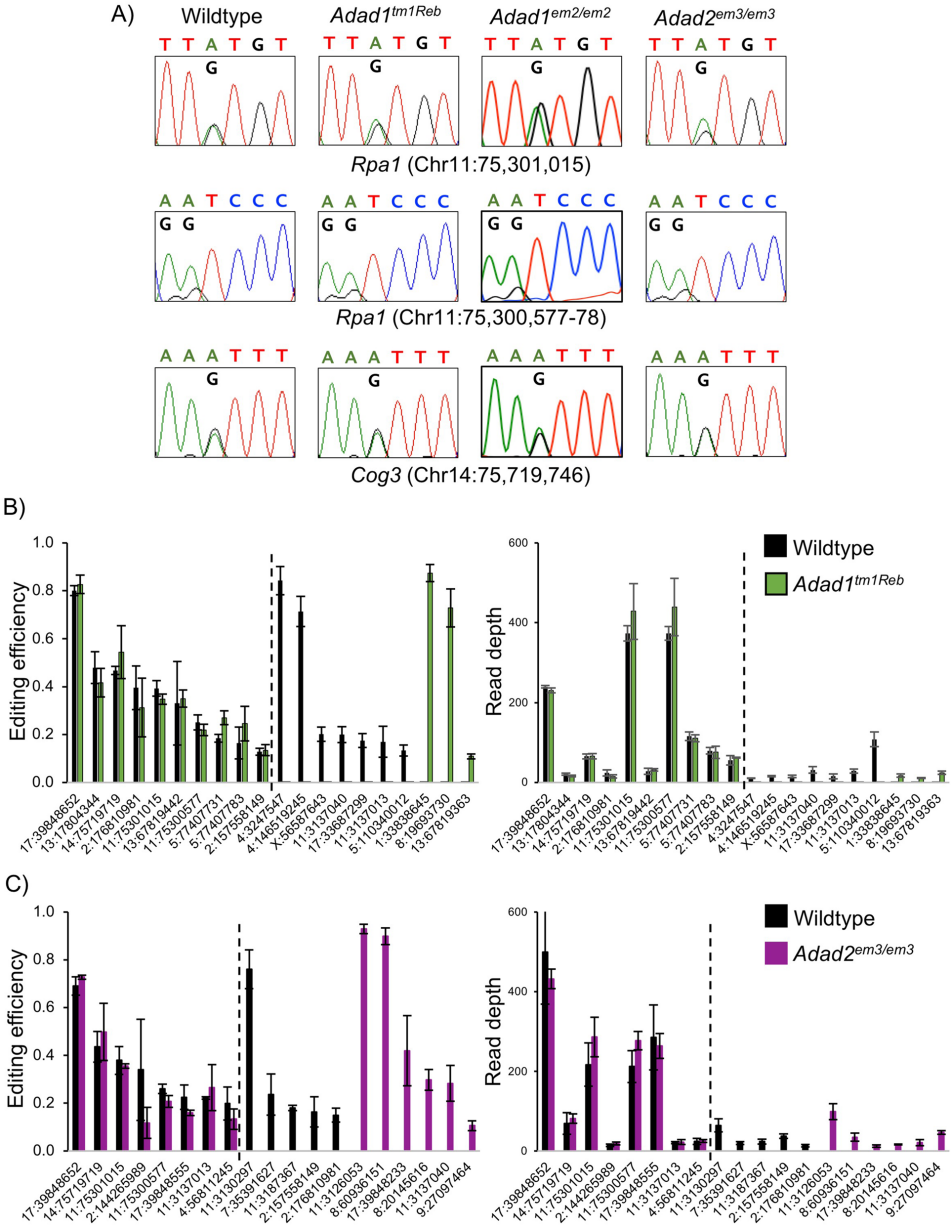
Mutation of *Adad1* and *Adad2* does not impact RNA editing at known germ cell RNA editing sites nor site selection or efficiency across the transcriptome. (**A**) Sanger sequencing chromatographs of known germ cell A-to-I RNA editing sites in adult wildtype, homozygous *Adad1* mutant, and homozygous *Adad2* mutant testes. All *Rpa1* sites detected in both spermatocytes and spermatids. *Cog3* site detected only in spermatocytes. Mixed A/G peaks indicate A-to-I editing sites. No significant changes in peak height were observed in mutant versus wildtype testes. N ≥ 3. Representative chromatographs shown. (**B**) and (**C**) RNA editing efficiency (fraction of edited reads) at all detected sites in (**B**) *Adad1* wildtype and mutant and (**C**) *Adad2* wildtype and mutant. Read depth (number of informative reads) for each site shown. Dashed lines demarcate genotype-independent and genotype-dependent events. Only sites detected in all three replicates of a given genotype were considered for analysis. N = 3, error bar—standard deviation.

RNA editing in the testis is rare and cell-type dependent, with fewer detectible RNA editing sites observed in germ cells as compared to the somatic cells of the testis^19^ suggesting one potential mode of RNA editing regulation in the male germ cell is active suppression. Given the germ cell-specific expression of both ADAD1 and ADAD2, analysis of RNA editing in *Adad* mutants focused first on known germ cell RNA editing sites observed primarily in spermatocytes and spermatids and a site observed exclusively in spermatocytes^19^. If either ADAD1 or ADAD2 regulated RNA editing in a manner similar to that observed for cADR-1, dramatic changes in the efficiency of editing at a given site would be expected. In contrast to this expectation, Sanger sequencing of bulk amplified RNA editing sites demonstrated very similar levels in the ratio of edited to non-edited targets across four known germ-cell RNA editing sites (Fig. 6A). However, Sanger sequencing of bulk amplicons is unlikely to detect subtle changes in RNA editing efficiency. Further, the ADADs may play an unexpected role in RNA editing site selection or impact RNA editing events outside those selected for Sanger sequencing. In order to address this, high throughput RNA sequencing of total RNA from late juvenile wildtype, *Adad1*^*tm1Reb*^, and *Adad2*^*em3*^ mutant testes was performed and RNA editing events in coding regions identified and quantified (Fig. 6B, C). As in previous reports, relatively few high confidence RNA editing sites were detected. For sites detected in both wildtype and mutant testes no alteration of RNA editing efficiency was observed supporting the Sanger sequencing analyses. However, for both *Adad1*^*tm1Reb*^ and *Adad2*^*em3*^, half of the detected RNA editing sites were detected in either wildtype or mutant testes, but not both. Further, these genotype-dependent RNA editing events had editing efficiencies similar to the genotype-independent events suggesting the ADADs may play a role in RNA editing site selection. However, this phenomenon could also be a result of artifacts from unusually low gene expression that can make it difficult to confidently parse real RNA editing events from sequencing or alignment errors. This difficulty is common to RNA editing identification pipelines as RNA editing generally occurs at a low frequency in most targets resulting in a low signal to noise ratio^27^. To determine if this was the case, read depth at each genotype-dependent site was quantified and compared to read depth at genotype-independent sites. This analysis showed that on average, read depth of genotype-independent sites was significantly higher than genotype-independent sites (5.2 times higher in the *Adad1* and 4.8 times higher in the *Adad2* datasets, respectively). Based on these observations, genotype-dependent RNA editing events likely represent RNA editing identification artifacts stemming from low gene expression as opposed to genuine events. These data demonstrate *Adad* mutation does not appreciably impact either RNA editing efficiency or site selection in coding regions.

## Discussion

Adenosine deaminase domain proteins are canonically associated with A-to-I RNA editing, whether it be as direct catalyzing enzymes or indirect regulators. The testis, and especially the male germ cell, is a site of relatively low RNA editing, leaving open the question of whether RNA editing is specifically repressed in this tissue. The aim of this work was to test whether two male germ cell-specific AD domain proteins, ADAD1 and ADAD2, regulate RNA editing and whether one, ADAD2, is required for male germ cell differentiation. Both ADAD1 and ADAD2 were shown to have amino acid mutations similar to other AD domain proteins that indirectly regulate RNA editing. However, mutation of either *Adad1* or *Adad2* resulted in no impact on RNA editing efficiency at known germ cell RNA editing sites. Additionally, no changes in testicular RNA editing efficiency or editing frequency were observed when assessed transcriptome-wide. Taken together, these data demonstrate neither ADAD1 nor ADAD2 regulate RNA editing in the testis suggesting other factors may be involved. Further, this work demonstrates both ADAD1 and ADAD2 play essential, and likely distinct, roles in germ cell biology.

As AD-domain proteins, both ADAD1 and ADAD2 represented reasonable targets for regulators of germ cell RNA editing. Yet, in both cases mutation resulted in minimal impacts on editing suggesting both proteins impact germ cell biology through some other mechanism. For a discussion of potential RNA editing considerations in the testis, please see the accompanying Supplementary Discussion. Non-RNA editing functions for AD-domain containing proteins is not without precedence. ADAR1, in particular, appears to impact the transcriptome via a number of mechanisms. As a result of interactions with other RNA bindings proteins ADAR1 has been shown to alter RNA stability^28^, RNA splicing^29^, miRNA processing^30^, and RNA storage^31^. In each case, these impacts were shown to be independent of ADAR1′s RNA editing capacity but generally involved direct RNA binding by ADAR1 suggesting AD-domain containing proteins generally function by regulating cellular RNAs. In the context of the testis, RNA regulation is especially important and it is reasonable to propose both ADAD1 and ADAD2 are required for germ cell differentiation due to their regulation of some other aspect of germ cell RNA processing.

For both ADAD1 and ADAD2, loss results in relatively minor gene expression changes in spite of pronounced phenotypes suggesting they both function post-transcriptionally. For ADAD1, this conclusion is buoyed by previous observations demonstrating it binds to the 3′ UTR of the protamine 1 (*Prm1*) mRNA^17^. *Prm1* is among the classically defined translationally regulated transcripts in the testis^32^ and proteins that bind to its 3′ UTR are often associated with translational regulation or RNA storage. However, mutation of *Adad1* did not impact *Prm1* translation or the translation of several other translationally regulated transcripts as reported by Connolly et al.^18^ suggesting ADAD1 may post-transcriptionally regulate a different set of transcripts.

Further supporting the argument that ADAD1 and ADAD2 are involved in post-transcriptional regulation, both have been identified as components of the chromatoid body (CB)^25^, one of multiple non-membrane bound cytoplasmic granules composed of RNA and RBPs found in germ cells^33^. Components of non-sense mediated decay^34^, miRNA, and piRNA pathways^35^ localize to the CB along with a wide range of RNAs and RBPs of unknown function. As a result of the RNAs and RBPs associated with it, the CB acts as a hub of post-transcriptional RNA regulation. The localization of ADAD2, in particular, favors a possible role in CB biology. CBs are thought to form from small, perinuclear precursor granules which appear late in pachytene spermatocyte differentiation^35^ and are marked by DDX25 ^36^, a pattern that very closely mimics ADAD2′s localization. Surprisingly, chromatoid body formation, as assessed by DDX25 and SMG1 localization, did not appear to be significantly altered in *Adad2* mutant spermatocytes. Further, ADAD2 granules only showed partial overlap with DDX25 in late pachytene and diplotene spermatocytes suggesting the ADAD2 granule has a role in mid- to late-stage spermatocytes distinct from CB formation and function. However, it should be noted, the overall composition of the CB in mutant round spermatids was not assessed, leaving open the possibility that ADAD2 regulates the early steps of CB protein acquisition. This argument is supported by the observation that ADAD2 granules are observed prior to DDX25 granules, ADAD2 and DDX25 co-localization is much higher during the early phase of DDX25 granule formation, and *Adad2* mutant germ cell differentiation defects coincide with those observed in DDX25 mutants^37^. Due to technical constraints, the impact of ADAD1 loss on CB formation was not assessed and will be the topic of future work.

In total, this report demonstrates ADAD1 and ADAD2 to be AD-domain containing proteins that do not regulate RNA editing but are required for normal male germ cell differentiation. These analyses also suggest ADAD1 and ADAD2 regulate post-transcriptional events but play distinct roles in germ cell differentiation, with ADAD2 functioning primarily in mid- to late-stage spermatocytes and ADAD1 likely mediating events in post-meiotic round or elongating spermatids. Future work using the alleles generated will aim towards fully characterizing the molecular roles of ADAD1 and ADAD2 in meiotic and post-meiotic germ cell RNA biology.

## Methods

### Protein domain analysis

For adenosine deaminase domain comparisons across species, A to I editase domain sequence was extracted from Uniprot (www.uniprot.org) for each protein of interest and aligned via ClustalW, using a Gonnet matrix, open gap penalty = 10, extend gap penalty = 0.2, and delay divergent = 30%.

### RNA extraction and quantitative RT-PCR

Tissues were collected from wildtype and mutant adult and embryonic tissues and flash frozen prior to RNA extraction. For *Adad* mutant models, testes were collected from *Adad1*^*tm1REB*^, *Adad1*^*em2*^, and *Adad2*^*em3*^, and wildtype males and flash frozen. Total RNA was isolated by Trizol Reagent (Ambion) as per manufacturer’s recommended methods. Extracted RNA was DNase I (Qiagen) treated, purified by RNeasy mini column purification (Qiagen), and quality and quantity assessed by Nanodrop prior to cDNA synthesis by Superscript III RT (Life Technologies) with random hexamer priming per manufacturer’s suggestions. SYBR Green (Applied Biosystems, Power SYBR Green PCR mastermix) quantitative RT-PCR utilized the primers indicated in Supp. Table 2. Relative fold changes were calculated as previously^38^ using *Rps2* as the endogenous control.

### Antibody generation

Antibodies against ADAD1 and ADAD2 were generated commercially by ThermoFisher. For ADAD1, the standard 80-day guinea pig protocol with a synthetic peptide comprised of amino acids 583–605 was used. Two anti-ADAD2 antibodies were generated through the standard 90-day rabbit protocol utilizing a synthetic peptide from amino acid 543 to the C-terminus. A portion of the terminal bleed serum for each antibody was affinity purified commercially. For each immunization, two animals were used (Animal #1 and Animal #2). Antibody usage for images in this manuscript was as follows: ADAD1 western: anti-ADAD1 affinity purified, ADAD2 immunofluorescence: Anti-ADAD2 terminal bleed animal #1, and ADAD2 Western blot: anti-ADAD2 affinity purified animal #2. In all cases, immufluorescent signal and western blotting signal were consistent across all antibodies against a given target.

### Western blotting

Testes were collected from adult *Adad1*^*tm1REB*^*, Adad1*^*em2*^*, Adad2*^*em3*^, and wildtype male mice. Total protein was extracted by RIPA buffer (50 mM Tris–HCl pH = 8, 150 mM NaCl, 1% NP-40, 0.5% sodium deoxycholate, 0.1% SDS) with protease inhibitors (EDTA-free, ThermoScientific) and colorimetrically quantified using the DC protein assay (BioRad). One hundred μg protein per sample was electrophoresed on 10% acrylamide gels. Following wet transfer of proteins to PVDF membrane (BioRad) the proteins were detected by western blotting as per^39^ as follows: anti-ADAD1 1:500 (guinea pig), anti-ADAD2 1:2000 (rabbit), and GAPDH 1:4,000 (Cell Signaling Technology 2,118, rabbit). Secondary antibodies: Goat-anti-guinea pig 1:2000 (Invitrogen A18775) and goat-anti-rabbit 1:2000 (BioRad 172–1,019). Images were developed with ECL reagent (Thermo Scientific) and visualized using an Azure Biosystems C600 imager.

### Immunofluorescence

Immunofluorescence was performed as in^20^. Testes were dissected from adult mice and fixed overnight in 4% PFA before embedding in paraffin and sectioned to 4 μm. Sections were deparaffinized by xylenes and rehydrated. Antigen retrieval was performed by boiling slides in citrate solution pH 5.95 for 7 min. Primary antibodies were incubated overnight at room temperature and secondaries for one hour at room temperature. Antibody concentrations were as follows: ADAD2 1:200. Secondary antibody: Alexafluor 488 goat-anti-rabbit 1:1,000 (Invitrogen A-11008). Samples were visualized on a custom-built microscope (Zeiss) with fluorescent and brightfield capabilities.

### Generation of CRISPR alleles

All animal use protocols were approved by both the Jackson Laboratories and the Rutgers University animal care and use committees. Mouse procedures were conducted according to relevant national and international guidelines (AALAC and IACUC). CRISPR-guide design utilized the guide design resources available from https://crispr.mit.edu/. This platform was used to obtain candidate sgRNA sequences with low offtarget specificity. Guide efficiency scores were calculated using https://www.broadinstitute.org/rnai/public/analysis-tools/sgrna-design and guides were selected based on the least number of off-target genes and highest efficiency scores. Preparation of sgRNA was as previously described^40^. Briefly, sgRNA templates were amplified with T7 promoter sequence-conjugated primers and transcribed with MEGAshortscript T7 Kit (Life Technologies) and purified by MEGAclear Kit (Life Technologies). The concentration of RNA was measured by a NanoDrop instrument (Thermo Scientific) and Agilent Bioanalyzer (Agilent Technologies, Inc) and stored at − 80 °C until used. C57BL/6 J and CB6F1/J mouse strains were used as embryo donors and pseudopregnant recipient dams, respectively. *Cas9* or Cas9-D10A nickase RNA (100 ng/μl; TriLink Biotechnologies and Sigma-Aldrich respectively), sgRNA (50 ng/μl) were injected into the pronuclei of zygotes. Oviduct transfers were performed on the same day into pseudopregnant dams. The resulting offspring were screened for mutations in the targeted regions. In each case, three alleles were obtained and each founder was used to generate homozygous male carriers. Each was analyzed for molecular ablation on the protein level by western blotting of testis tissue and a single allele selected for each gene.

Genotyping of generated alleles utilized primers described in Supp. Table 2. PCR conditions for *Adad1*^*em2*^ were as follows: a 30 s primer melting step at 95 °C, 15 s anneal at 56 °C and a 60 s elongation at 72 °C, repeated 37 times with a final 5-min elongation at 72 °C. *Adad1*^*tm1REB*^ genotyping PCR consisted of separate reactions for wildtype and mutant alleles. Wildtype conditions were a 30 s primer melting step at 95 °C, a 15 s anneal at 58 °C and a 2 min elongation at 72 °C, repeated 40 times with a final 5-min elongation at 72 °C. Mutant reaction conditions consisted of a 30 s primer melting step at 95 °C, a 15 s anneal at 56 °C and a 60 s elongation at 72 °C, repeated 37 times with a final 5-min elongation at 72 °C. *Adad2*^*em3*^ genotyping conditions consisted of a 30 s primer melting step at 95 °C, 30 s anneal at 67.4 °C and a 30 s elongation at 72 °C, repeated 35 times with a final 5-min elongation at 72 °C.

### Histological and sperm parameter evaluation

For basic histological analyses, testes and epididymides from adult animals of the indicated genotype were collected, fixed overnight, and embedded in paraffin. Four μm sections were used for all analyses. For analysis of retained spermatids, testes from adult *Adad1* wildtype and mutant mice and fixed overnight in Bouin’s fixative. Sections were stained with periodic acid–Schiff’s reagent. Three cross-sections at least 100 μm apart were scored per animal. Stages were determined based on the criteria set out in^41^ and retained spermatids were counted if morphologically mature spermatozoa were detected in stages IX or X. Sperm concentration was determined by mincing epidydimal tissue in 1 ml of phosphate-buffered saline (PBS) and incubating for an hour at 37 °C. Samples were diluted 1:10 in PBS and sperm quantified as previously^39^. The remaining sample was centrifuged at 100×*g* at room temperature for 5 min to remove debris and applied to charged slides for sperm morphology analysis. Slides were incubated overnight in a humid chamber, air-dried, and stained by PAS. Sperm morphology parameters were applies as in^18^. To quantify tubules with round spermatids, testes from *Adad2* males were fixed overnight in 4% PFA as described above, deparaffinized, rehydrated, and stained with DAPI Fluoromount-G (Southern Biotech). Three cross-sections at least 100 μm apart were scored per animal. All images were captured on a custom-built microscope (Zeiss) with fluorescent and brightfield capabilities.

### Fertility screens

Fertility of *Adad1*^*tm1REB*^*, Adad1*^*em2*^*,* and wildtype males was assessed by mating adult mutant males and littermate control males with fertile C57BL/6 J females (6–8 weeks old). Litter number and size was recorded for a period of 4 months.

### Sanger sequencing of known germ cell RNA editing sites

RNA was isolated from adult *Adad1*^*em2*^*, Adad1*^*tm1REB*^*, Adad2*^*em3*^*,* and wildtype testes as described above and reverse transcribed into cDNA using the SuperScript III kit (Invitrogen). Standard PCR was utilized for site-specific amplification using the primers indicated in Supp. Table 2. Samples were then sequenced via standard Sanger sequencing. For all editing sites analyzed, concurrent genomic DNA was sequenced from the same individuals to confirm adenosine encoding at selected site.

### RNA-sequencing and transcriptome-wide RNA editing analysis

RNA sequencing was conducted as outlined previously by Snyder et al.^23,42^. Briefly, individual samples were collected into RNAlater and send for paired-end RNA sequencing on an Illumina HiSeq 2000 at The Jackson Laboratory (Bar Harbor, ME). Total RNA extraction via the mirVana RNA isolation kit (Life Technologies, Grand Island, NY) was performed per manufacturer’s recommendation, including DNase treatment. RNA sequencing libraries for 100 bp paired-end sequencing were produced using the TruSeq RNA Sample prep Kit v2 Set A and B (Illumina, San Diego, CA). For *Adad2* mutant gene expression, gene and isoform abundance were calculated as a function of an extended testis transcriptome^20^ within individual samples by RSEM^43^. Significant differences were calculated by EBSeq and plotted using ggplots2. *Adad2* mutant RNA-sequencing data has been deposited in GEO under the accession GSE150755. For cell-type expression profiles of differentially expressed genes, single-end 76 bp RNA-seq strand-specific reads derived from isolated testicular cell types were obtained from the SRA database (GEO accession numbers GSE43717, GSE43719, and GSE43721^24^). Individual samples were aligned to the expanded transcriptome and expression estimated via RSEM. Expression estimates were extracted for differentially expressed genes and the values plotted by heatmap using ggplots2. Assessment of RNA editing was performed as previously described by Snyder et al.^19^.

## Supplementary information

Supplementary information.
