# Supplementary material for: ADAD1 and ADAD2, testis-specific adenosine deaminase domain-containing proteins, are required for male fertility
Source: Sci Rep. 2020 Jul 14;10:11536. doi: 10.1038/s41598-020-67834-5 (PMC7360552; doi:10.1038/s41598-020-67834-5)
Supplement: Supplementary file 1 — Supplementary information. [file 41598_2020_67834_MOESM1_ESM.pdf]

Supplementary Information for:

ADAD1 and ADAD2, testis-specific adenosine deaminase domain-containing proteins, are required for male fertility

Elizabeth Snyder*[1,21], Lauren Chukrallah[12], Kelly Seltzer[13], Leslie Goodwin[24], Robert E. Braun[25]

[1] Rutgers University, the State University of New Jersey, New Brunswick, NJ
[2] The Jackson Laboratory, Bar Harbor, Maine

* Corresponding author

[1] Elizabeth Snyder: elizabeth.snyder@rutgers.edu
[2] Lauren Chukrallah: lauren.chukrallah@rutgers.edu
[3] Kelly Seltzer: kduym@sebs.rutgers.edu
[4] Leslie Goodwin: Leslie.Goodwin@jax.org
[5] Robert Braun: bob.braun@jax.org

SUPPLEMENTARY DISCUSSION

Special considerations regarding testicular RNA editing

Multiple factors have been identified as regulators of RNA editing with the best studied being the AD-domain proteins themselves. This report and previous related work (19) shows that while germ cell RNA editing is catalyzed by a known RNA editing enzyme, other AD-domain containing proteins do not appear to regulate germ cell RNA editing. These findings suggest other mechanisms of control may be at play. While *cis* elements within RNA editing targets have dramatic impacts on RNA editing efficiency by giving rise to specific RNA secondary structures (28), it seems unlikely that *cis* elements within the germ cell transcriptome alone explain the relative lack of RNA editing. This is especially true given the complexity of the male germ cell transcriptome exceeds even that of the brain (20, 24), a site of extremely high RNA editing levels. However, RNA editing has been shown to be quite sensitive to a cell's complement of RNA binding proteins (RBPs) (29) and male germ cells express an incredibly wide range of RBPs (30), in part due to their tight reliance on post-transcriptional processes for normal differentiation (21). RBPs, which drive an extremely diverse range of biological process, act on the transcriptome and as such can change the availability, structure, localization, and stability of RNA editing targets in a cell-type or tissue-dependent manner. Which, if any, germ cell-specific RBPs interact with the RNA editing pathway remains an open question, however efforts leveraging RNA-sequencing data from large-scale projects like ENCODE may provide valuable insight.

In addition to individual RBPs, it has been demonstrated that entire RNA processing pathways intersect and impact RNA editing. In particular, splicing has been shown to have a robust influence on RNA editing for a large number of targets (29). The interaction of these two pathways is reasonable given RNA editing is known to occur co-transcriptionally (7) and is often the result of exonic and intronic *cis* elements interacting to form the double stranded RNA template used by ADAR enzymes (30). Consistent with this, a recent report demonstrated that global reduction of splicing leads to significant increases in editing efficiency (31) while mutation of specific alternative splicing factors altered editing of more specific targets, with decreased splicing generally being associated with increased editing. These

findings are buoyed by the observation that a large number of alternative splicing proteins have been identified as RBP regulators of RNA editing (27). The interaction of splicing and RNA editing may be especially important in the context of the male germ cell where alternative splicing is particularly high (20), which mechanistically may serve to globally repress RNA editing. To facilitate the depth of alternative splicing observed, the testis expresses a wide range of splicing factors, some or many of which may influence total or site-specific RNA editing. As germ cells develop, they leverage different suites of alternative splicing programs (32) to facilitate cell-specific alternative splicing patterns across germ cell development. This cell-specific splicing regulation will require careful dissection to identify potential splicing regulators of germ cell RNA editing.

Phenotypic differences between *Adad1*^{tm1Reb} relative to *Adad1*^{em2} may be driven by multiple mechanisms

While it is possible the difference in phenotype between *Adad1*^{tm1Reb} and *Adad1*^{em2} may be due to differences between wildtype ADAD1 and the abnormal proteins detected in the *Adad1*^{tm1Reb} testes, it seems most likely the reduced phenotypic severity in *Adad1*^{tm1Reb} relative to *Adad1*^{em2} is due to a reduction, but not total loss, of functional ADAD1 protein in *Adad1*^{tm1Reb}. Further, this outcome may be exacerbated by a number of other mechanisms. As with all CRISPR generated alleles, it is feasible carrier mutations in the background of the CRISPR allele are leading to a more severe phenotype. However, this is extremely unlikely as extensive analyses confirmed no carrier mutations at other probable CRISPR targets within the genome. A much more likely mechanisms is that the two alleles behave differently as a function of their slightly different genetic context. Although both mutant models were derived from the same strain, due to breeding the two alleles were analyzed on slightly different genetic backgrounds. This hypothesis is supported by previous observations (18) showing that when moved to a different genetic background, the *Adad1*^{tm1Reb} fertility phenotype changed dramatically. These suggest ADAD1 function may be reliant on genetic modifiers and open the possibility of defining ADAD1's molecular function via genome-scale modifier screens.

Supplemental Table 1. Average expression of select germ cell RNA binding proteins in isolated testicular cell types. Values represent average TPM ± standard deviation. Bold and italic indicates highest average TPM by cell type.

	Cell type				
Gene Name	Sertoli	Spermatogonia	Spermatocyte	Spermatid	Spermatozoa
Adad1	0.02 ± 0.02	4.31 ± 0.42	207 ± 3.18	***261.28 ± 2.51***	0.39 ± 0.21
Adad2	0 ± 0	2.2 ± 0.24	***29.61 ± 2.27***	4.19 ± 0.3	0.3 ± 0.19
Dazl	0.03 ± 0.01	***66 ± 2.16***	44.43 ± 2.93	30.42 ± 2.11	0.09 ± 0.01
Ddx4	0.84 ± 0.2	52.42 ± 0.86	***405.02 ± 11.56***	292.23 ± 2.95	0.9 ± 0.14
Ddx25	0.5 ± 0.18	11.47 ± 0.58	***753.56 ± 8.56***	583.52 ± 4.26	1.28 ± 0.2
Dicer1	13.41 ± 0.1	41.27 ± 0.36	2.52 ± 0.08	2.2 ± 0.14	***59.05 ± 1.09***
Mael	0.08 ± 0.03	45.73 ± 2.55	***1045.46 ± 15.2***	857.8 ± 20.75	1.54 ± 0.46
Rnf17	0.78 ± 0.17	15.24 ± 0.53	87.27 ± 5.34	***112.07 ± 1.48***	1.47 ± 0.11
Piwil1	0 ± 0	0.69 ± 0.07	***278.61 ± 9.23***	18.3 ± 0.02	0.07 ± 0.08
Piwil2	0.24 ± 0.04	23.45 ± 0.77	***68.08 ± 0.79***	14.52 ± 0.66	3.98 ± 0.34
Trdr1	0.04 ± 0.02	10.19 ± 0.22	***77.69 ± 1.15***	8.37 ± 0.38	0.48 ± 0.06
Ybx2	0.05 ± 0.06	3.18 ± 0.63	***2813.62 ± 396.52***	2179.9 ± 64.64	30.82 ± 2.73
Ybx3	163.32 ± 2.74	341 ± 3.43	***2946.55 ± 68.63***	2104.59 ± 5.13	48.15 ± 1.35

Supplemental Fig. 1

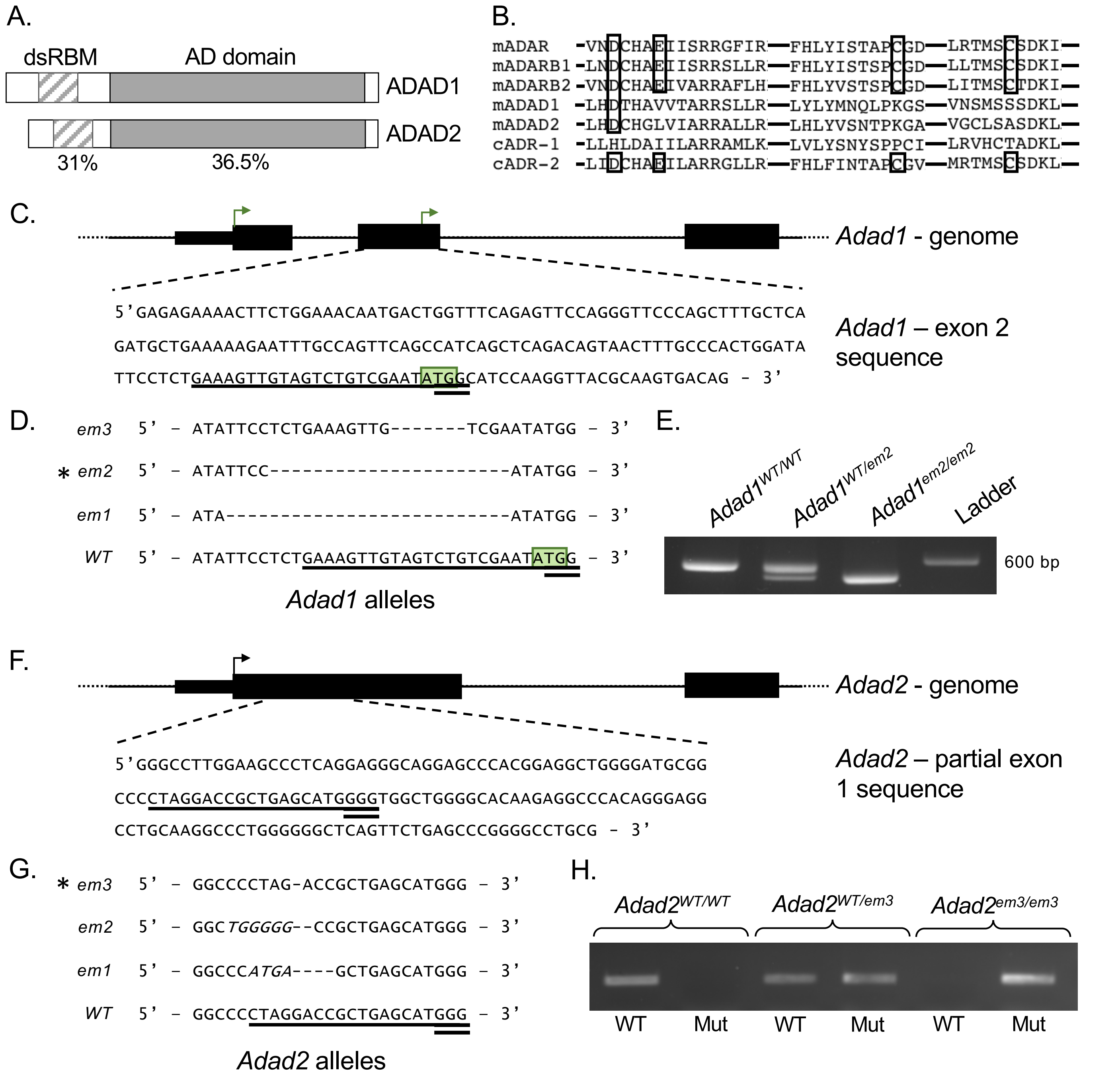

CRISPR-*Cas9* targeting of *Adad1* and *Adad2.* A. Protein maps of ADAD1 and ADAD2 indicating dsRNA binding domain (dsRBM) and adenosine deaminase (AD) domain. Domain level similarities for ADAD2 relative to ADAD1 indicated as percentages. **B.** Comparison of amino acid sequences of select AD domain proteins. Conserved zinc-coordinating residues shown in boxes. **C.** *Adad1* genomic locus. Large boxes represent coding regions, small box indicates 5’ UTR, green arrows indicate potential translation start sites. *Adad1* exon 2 sequence and CRISPR targeting scheme. gRNA sequence underlined, PAM site double underlined, and 3’ translation start site boxed in green. **D.** *Adad1* CRISPR mutant alleles. Dashes indicating deleted nucleotides. Molecular impacts are as follows: em1 – 29 bp deletion starting at chr3:37,064,304, em2 - 22 bp deletion starting at chr3:37,064,309, and em3 – 7 bp deletion starting at chr3:37,604,320. * indicates allele selected for further study. gRNA, PAM site, and 3’ translation start site indicated in WT sequence. **E.** PCR-based genotyping of *Adad1* CRISPR allele selected for further study showing detection of 22 bp deletion. **F.** *Adad2* genomic locus with partial exon 1 sequence and CRISPR targeting scheme. Large boxes represent coding region, small box indicates 5’ UTR, black arrow indicates only known translation start site. Within sequence gRNA sequence underlined, PAM site double underlined. **G.** *Adad2* CRISPR mutant alleles with dashes indicating deleted nucleotides. Molecular impacts are as follows: em1 – 8 bp deletion deletion starting at chr8:119,612,902 plus a 4 bp insertion (ATGA), em2 – 8 bp deletion starting at chr8:119,612,900 and a 6 bp insertion (TGGGGG), and em3 – 1 bp deletion at chr8:119,612,906. * indicates allele selected for further study. gRNA, PAM site, and 3’ translation start site indicated in WT sequence. **H.** PCR-based genotyping of selected *Adad2* CRISPR allele using allele specific priming to detect the single nucleotide deletion. WT – primers specific to the wildtype allele. Mut – primers specific to the CRISPR allele. Genotypes of each sample indicated above.

Supplemental Fig. 2

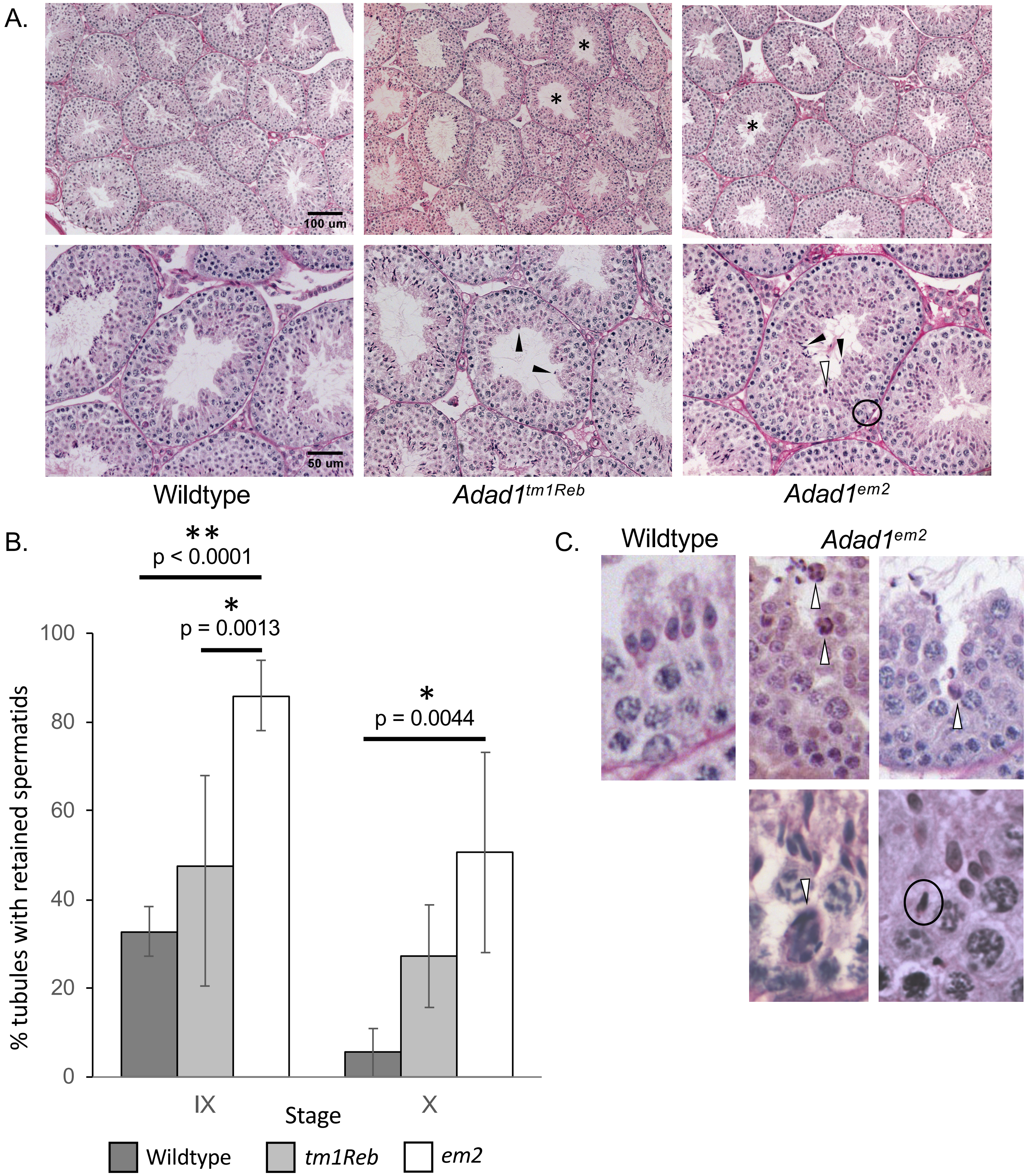

Testicular histology of *Adad1* homozygous mutants demonstrate a minor spermiation defect with *Adad1* mutation. A. Comparison of wildtype and *Adad1* homozygous mutant adult testis histology stained with PAS. Scale indicated by bar. Upper panels: asterisks indicate tubules with retained spermatids. Lower panels: retained spermatids indicated by arrow heads, spermatids undergoing resorption indicated by empty arrow heads and circles. **B.** Quantification of tubules with retained spermatids by tubule cross-section stage in wildtype (WT) and homozygous mutant adult testes. *tm1Reb* - *Adad1*^{tm1Reb}. *em2* - *Adad1*^{em2}. N ≥ 4, error bars – standard deviation, significance indicated. **C.** High magnification images of late stage tubules with retained spermatids. Wildtype shown for comparison. Spermatids undergoing resorption indicated by empty arrow heads and circles.

Supplemental Fig. 3

A.

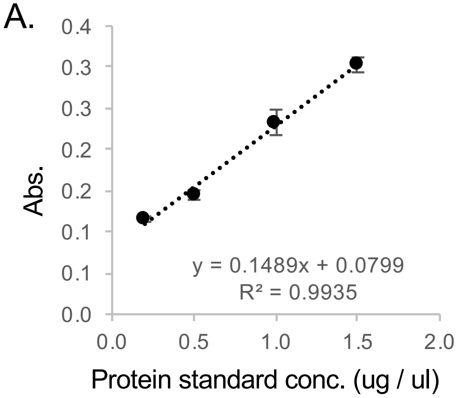

	Average Abs. (corrected by dilution factor)	Est. protein conc. (ug/ul)	Dilution factor (5 ug / ul)
Brain	1.67	10.69	0.47
Heart	1.76	11.25	0.44
Liver	2.28	14.78	0.34
Lung	1.65	10.56	0.47
Muscle	0.93	5.68	0.88
Ovary	1.09	6.76	0.74
Testis	1.42	9.01	0.55

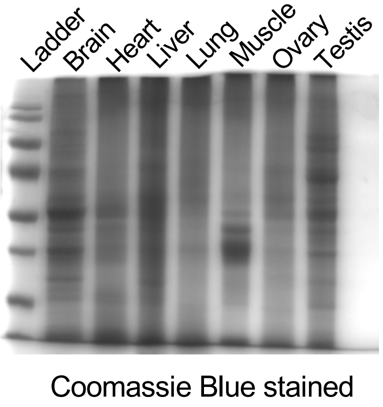

B.

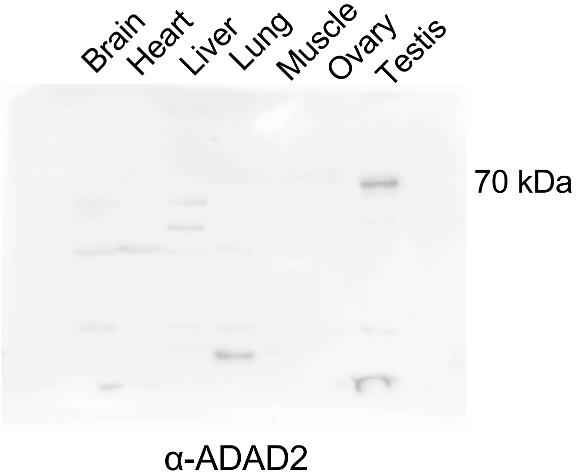

C.

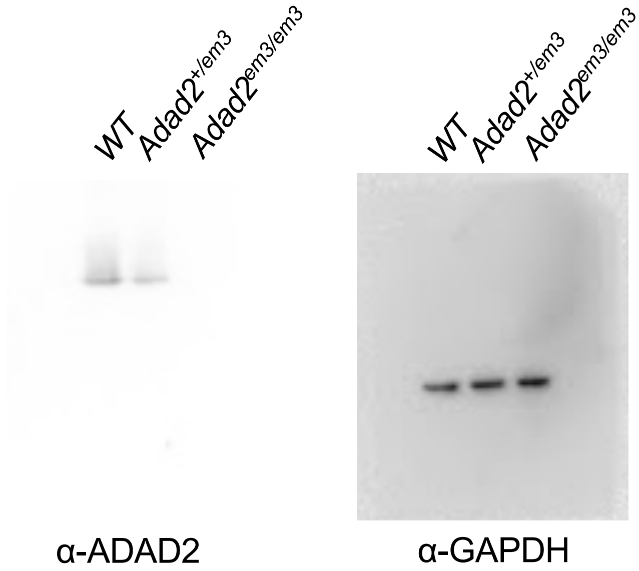

D.

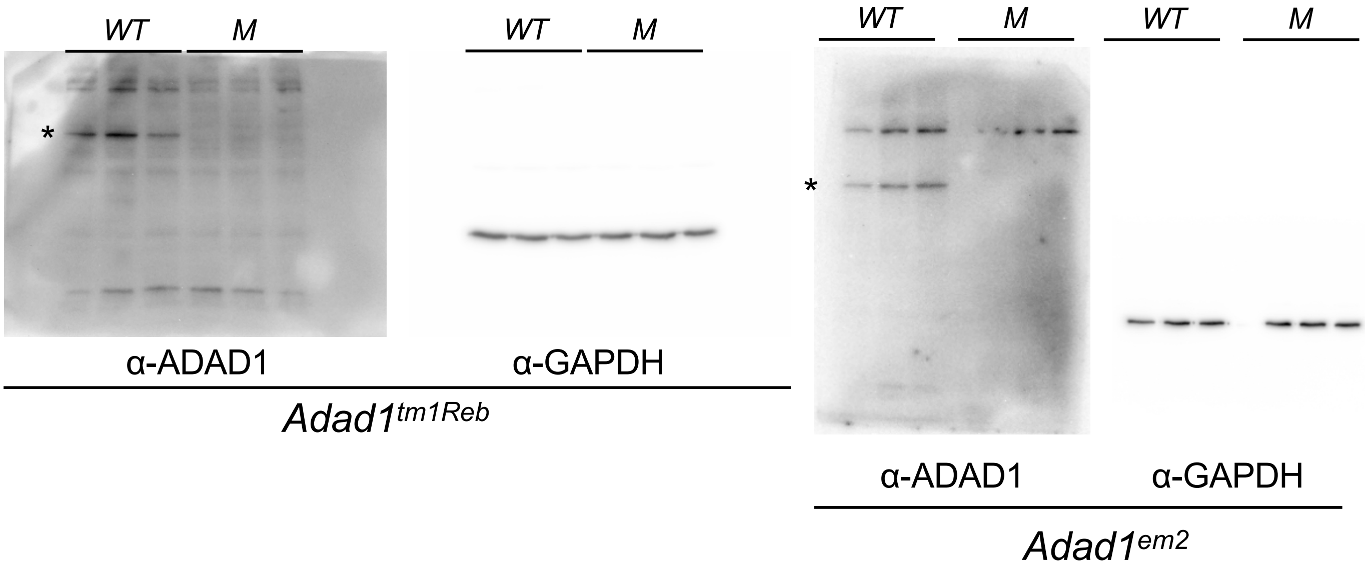

Supplementary information regarding Western blotting results. A. Protein quantification for tissue panel by DC assay including establishment of standard curve, estimation of protein concentration by sample, and confirmation of equivalent loading by Coomassie staining. Full length western blots from **B.** Figure 1D, **C.** Figure 2D, and **D.** Figure 2B. Antibody probe indicated below each blot and the associate genotype (WT – wildtype, M – mutant) or tissue indicated. ADAD1 indicated with an asterisk.

Supplemental Fig. 4

A.

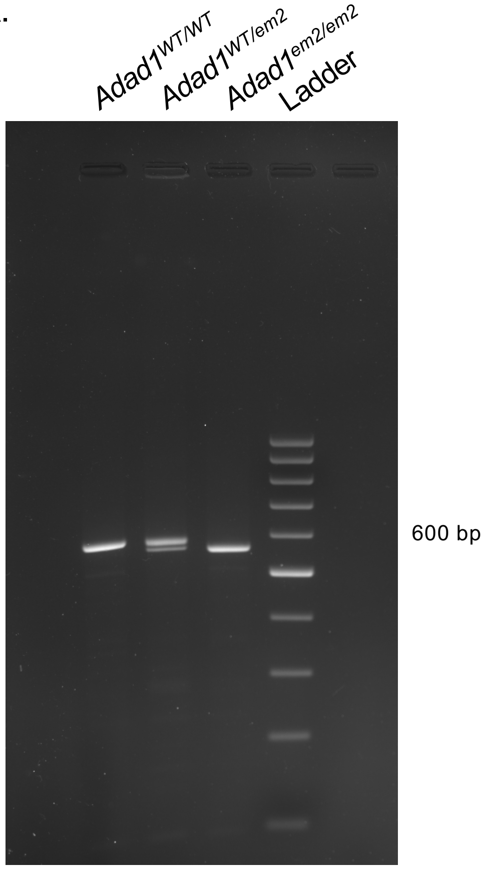

B.

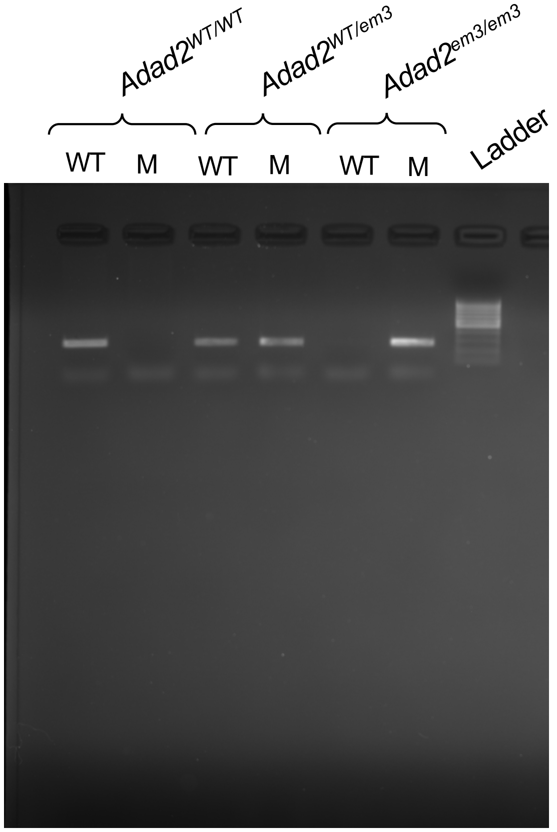

Full length DNA gels from A. Supp. Figure 1E and **B.** Supp. Figure 1H. Genotype (WT – wildtype, M – mutant) indicated.

Supplemental Table 2. Primers used in this study with chromosomal locations. For genotyping primers, the target allele is indicated in parenthesis.

Target	Experiment	Forward	Chromosome location:	Reverse	Chromosome location:
Adad1	qRT-PCR	TACAGGGAGCCTTGCTGAGT	Chr3:37,085,098-37,085,117	TGATGTGAGTGCGTCATCAA	Chr3:37,091,914-37,091,933
Adad2	qRT-PCR	CATGGCTAACCTGGGTGTCT	Chr8:119,613,083-119,613,102	TCTGGTCTCTCCAGCTGCTT	*
Rps2	qRT-PCR	CTGACTCCCGACCTCTGGAAA	Chr17:24,721,664-24,721,684	GAGCCTGGGTCCTCTGAACA	Chr17:24,721,844-24,721,863
Adad1 (WT)	Genotyping	TGACCGCTCTAGGTTGTCCT	Chr3:37,063,268-37,063,287	GGTCAGTCGCGTCTTTCAA	Chr3:37,063,653-37,063,671
Adad1 (tm1Reb)	Genotyping	CCGCTATCAGGACATAGCGTTGGC	Chr3:37,064,066-37,064,085	CCCATGGCTACAAACAATCC	**
Adad1 (CRISPR)	Genotyping and Sanger sequencing	GGTTTGGTTCTGTCCCCTCTTG	Chr3:37,063,863-37,063,884	ACATACACCCACACCCACATCAC	Chr3:37,064,431-37,064,453
Adad2 (WT)	Genotyping	TGGGGATGCGGCCCCTAGG	Chr8:119,612,888-119,612,906	CTGCAGTCTGGTCCTCCAAAAAGG	Chr8:119,613,109-119,613,132
Adad2 (CRISPR)	Genotyping	TGGGGATGCGGCCCCTAGA	***	CTGCAGTCTGGTCCTCCAAAAAGG	Chr8:119,613,109-119,613,132
Adad2 (CRISPR)	Sanger sequencing	GAAAAAGAGCGCGGGGACG	Chr8:119,612,747-119,612,765	CTGCAGTCTGGTCCTCCAAAAAGG	Chr8:119,613,109-119,613,132
Rpa1 - site 1 editing	Editing analysis	CTCAGAGGGCTGTGTGTGAA	Chr11:75,301,112-75,301,141	AGACAAAAAGGTGCCACCAC	Chr11:75,300,928-75,300,947
Rpa1 - site 2/3 editing	Editing analysis	TGATAAGCACTGGCTTGCTG	Chr11:75,300,658-75,300,677	AACCGAACGGTAACTGATGC	Chr11:75,300,457-75,300,476
Cog3 - mRNA editing	Editing analysis	CACAGACGACGATCTCTCCA	Chr14:75,724,789-75,724,808	TGAACTCCTCCAGCTGCTCT	Chr14:75,716,876-75,716,895
Cog3 - genomic site	Editing analysis	GACTCGTTCTCGGAGCTTTG	Chr14:75,720,143-75,720,162	CTGTGCTGACACACCTGGAC	Chr14:75,719,629-75,719,648

* Recognizes a splice junction in the 3' end of the *Adad2* transcript.
** Recognizes the mutational insert sequence and is not found within the wildtype mouse genome.
*** Recognizes the *Adad2* CRISPR mutant allele as described in Supplemental Figure 1.
